# The role played by insider transfer information in esports coverage

**DOI:** 10.3389/fspor.2024.1346984

**Published:** 2024-04-16

**Authors:** Yi Rong, Jiaqi Li

**Affiliations:** Faculty of Humanities and Arts, Macau University of Science and Technology, Macau, Macau SAR, China

**Keywords:** insiders, esports information, esports industry, off-season, esports career

## Abstract

Esports is a rapidly growing industry and related information garners the attention of audiences both inside and outside the arena. The electronic competition system has become more structured over time and there are now standard off-seasons, which coincide tightly with an intensive period of player transfers. However, at the same time, the off-season has few events to cover and transfer information is highly confidential, so any insights regarding possible transfers are of deep interest to audiences. As a result, unofficial esports insiders often leak transfer information. To explore this phenomenon in greater depth, the methodology used in this study was based on the content analysis of information relating to 10-years in the life of the esport Counter-Strike: Global Offensive (CS:GO). Data was collected from the Perfect World Esports APP and the HLTV websites to analyze the informants and the development of transfer information and its influence. It was found that, during the off-season, insider transfer revelations became the primary focus of content in the esports media sector. As the number of viewers participating in discussions about potential transfers has grown, esports tipsters have discovered a new career path within the industry. A comprehensive assessment of the audience and professional practitioners reveals the essential qualities that esports insiders should possess to excel in this field and for their careers to develop.

## 1 Introduction

Esports have come a long way since their inception in the early 1970s. After 50 years of rapid development, advances in technology have enabled them to reach a mature stage of development. There is quite significant disagreement about the definition of esports, but, in a recent review of the esports literature, Formosa et al. (1) suggest they might be broadly characterized in the following way: “*Organized competitive digital gaming, played on a spectrum of professionalism—its organized and competitive aspects lead to elements often associated with, but not necessary for esports, including: spectators and fans; tournaments and leagues; training and skill development; and sponsorship, commercial partnerships, and prize money.*” (1: 227) The expansion of electronic competitions, the increasing variety of esports, and the growing frequency of events at all levels have prompted companies and practitioners in related industries to build an ecology that encompasses all aspects of the upstream, downstream, and backstage elements of the esports enterprise. As a result, esports and traditional sports are gradually merging. Thus, esports featured in the 2018 Asian Games in Jakarta and were assigned a medal spot in the 2022 Asian Games in Hangzhou (China) (2). The competition system for popular esports has reached a new stage of maturity, including ideal competition specifications and the establishment of competition brands with commercial value. According to the 2023 *Global Esports Industry Development Report* (3), the global esports audience was set to reach 478 million that year. Information of interest to this audience includes the events themselves, the agencies representing esports artists, the atmosphere around events, and any content related to esports derivatives.

Popular esports competitions are conducted throughout the year, with events occurring frequently within each game's particular season. There is also an off-season between the seasons that allows players to rest and the clubs to trade to strengthen their lineup. Esports news is typically similar to traditional sports news and encompasses things like event information, transfer information, review articles, and other relevant bits and pieces. Transfer information is part of the architecture of fandom, with a ritual and a routine that intensifies during transfer windows. Transfer information enables fans to engage with the game and with “their” club (4). Traditional sports such as football contain both real information and rumor in their handling of transfers and esports are no different in this respect (4, 5). However, esports informants (individuals who disseminate various information about esports) tend to stand outside the mainstream media, with the main protagonists being esports insiders who are active during the off-season as independent media commentators. As the kinds of information esports audiences are interested in are expanding to cover off-season activities, this is turning esports insiders from backstage to frontstage participants. The content of the information revealed by these parties has elicited mixed feedback from both official practitioners and audiences. Generally, it is felt that, as esports reporting is becoming increasingly popular and influential, it should be held to an accepted set of standard professional norms. However, to date, little research regarding esports reporting has been undertaken, making it difficult to know exactly what those norms should look like or how they should be enacted or enforced.

This paper uses a case study of a particularly popular esport, Counter-Strike: Global Offensive (CS:GO), to lay some foundations for addressing the above issue. The study undertook a content analysis of news released about CS:GO between 2013 and 2023, and explored the types of information being published, the sources of the information, whether it was released in the competition season or off-season, and its relative popularity. Particular attention was paid to news being released by esport insiders and the kind of impact it was having. The goal was to understand how the role of esports insider informants was developing, how audiences were engaging with the information they were publishing, and the ways in which the role might be evolving into a recognizable profession. As noted above, insider informants have an especially noticeable presence during the off-season, with one of the main topics they address being prospective transfers. For that reason, we take a particular interest here in transfer-related news and its impact.

In the next section, we provide some background regarding the esports industry and its characteristics. This is followed by a detailed description of our research objectives and the questions the study was seeking to address. Section 4 gives a detailed explanation of how we approached the study and conducted our analysis. After that, we present the results of the study and consider how they answer the questions we were posing, before engaging in a wider discussion of the potential implications of the results for the esports industry and the professional development of the role being played by insider informants.

## 2 Background

We have already noted that esports might be broadly defined as organized competitive digital gaming (1). However, despite the lack of consensus about a definition, Formosa et al. also found a number of features that recurred across many definitions, such as: “*spectators and fans; tournaments and leagues; training and skill development; and sponsorship, commercial partnerships, and prize money.*”(1: 227). Originally, esports served a promotional role, but quickly became attractive to consumers in their own right. Competitive events are more attractive to esports consumers than storytelling and gaming experiences (6). Witkowski describes playing esports as a “rich sensory experience that calls for layer upon layer of physically demanding action in order to be competitive in the high-performance game” (7). Esports tournaments are a collection of high-level events that are also a nexus of relationships, engaging all the actors involved in both the staging and realization of the experience, including gaming companies, players, communities, broadcasting stations, and sponsors (8). Thus, esports have had a big impact on the sports industry and their fanbases are constantly increasing. Athletes, professional teams, sponsors inside and outside the sport, and the media all play an important part in the promotion of the industry (9). Effective esports information is therefore crucial to the industry's development and is of importance to both professional practitioners and audiences. The delivery of esports information needs to meet the commercial interests of official bodies, such as event organizers, clubs, and game manufacturers, while keeping audiences informed with relevant event information. Within the esports industry, the exact ways in which information is classified and distributed are shaped around its tournament and season-based structure.

### 2.1 The relationship between esports and traditional sports

The esports industry revolves around tournaments. However, these tournaments differ from traditional sports events in meaningful ways. First of all, they differ in how information is communicated about them through media and platforms. Esports league information is mainly broadcast and disseminated through Internet platforms. This allows it to reach a broad global audience. In contrast, traditional sports leagues usually rely on TV and on-site venues (10). Second, in the traditional literature, conventional sports and esports are argued to have different characteristics. Traditional sports typically involve gross muscle exertion that emphasizes physical prowess and endurance. Esports, however, are less concerned with muscular exertion, emphasizing instead skills such as reaction speed, tactical strategy, and hand-eye coordination (11). It is argued that, in traditional sports, the outcomes of training and experience relate to players’ capacity to use different muscle groups. This ultimately determines the level they can reach. Esports competitors are selected according to factors such as hand-eye coordination and reaction capabilities, game comprehension, and ability to engage in tactical teamwork. However, it should be noted, that some researchers suggest that esports does involve physical exertion when competing (12–15) and that competitors often do undertake significant amounts of physical training (16). Third, esports competitions are based on video games, and their audience is primarily people who also play games, presenting a unique opportunity for brands to engage with a highly motivated and passionate audience (17). The audience for traditional sports is much more diverse and many who watch never actually play the sports themselves (18). Fourth, cultural and social concerns play a significant role in how esports are accepted. Despite being recognized as performance sports in the Asian Games, many cultures are still skeptical about their entitlement to this status, though others have moved to accept it (19). Most traditional sports have much more mainstream status in the cultures where they are practised.

Despite the above differences, esports also share many similarities with their traditional counterparts. First, both esports and traditional sports emphasize competitive spirit, teamwork, and individual performance (16). Second, both esports and traditional sports leagues are seen to have commercial potential, which attracts sponsors and advertisers, forming an independent business ecosystem (4, 20). Third, players and athletes in both sports can boast a robust fan base with dedicated supporters, audiences, and an individual and club-based fan culture (21). Finally, they both have a global reach, with international competitions attracting players and spectators from all corners of the world (22). Despite, their relatively new presence, esports competitions have been especially successful at garnering the potential of the Internet, which has given rise to an industry with global impact (10).

### 2.2 Esports and their competitive structure

As their development and planning have become perfected, esports events have come to take place throughout the year. Online esports events are geographically divided because of network technology restrictions. Offline tournaments, meanwhile, are regularly held in cities around the world. Given, these constraints, careful planning has played an important part in the popularization of esports (23). The classification of esports events is based on multiple factors, such as the scale of the event, the degree of influence, and the level of official recognition of the game. Esports come in different forms, and they are usually based on popular competitive multiplayer video games. Mainstream esports game categories include real-time strategy (RTS) games, first-person shooters (FPS), multiplayer online battle arena (MOBA) games, sports simulation games, fight technology games (FTG) (24). Different types of esports have different competitive rules (25). Statistics gathered from The Rankings suggest that Dota 2 is the most popular esports game. This is followed by Counter-Strike: Global Offensive (CS:GO), Fortnite, League of Legends, Pubg, and Honor of Kings. These games have all accumulated more than 50 million yuan in revenue since their release (26). Moreover, they all have mature competition planning and a stable tournament system, with the prize pool and number of events being crucial for their popularity. Below we give an overview of the four most popular games and their competitive structure:

#### 2.2.1 Dota 2

Dota 2 has two types of events: official and third-party events. Official events include international championships (e.g., Dota 2), which are held yearly. Regional Major Championships are held four times a year, with top teams from each region having to first pass through Minor qualifiers. The third-party tournaments include regional leagues, international cups, and masters tournaments. Entrance to competitions is according to a team's position, which is based on credits and qualification.

#### 2.2.2 Counter-strike: global offensive, CS:GO

The only official CS:GO tournament is the Major Championship, which is held twice a year. The tournament is co-organized by the game publisher, Valve, and a third-party tournament company. It is the biggest and most prestigious event. However, there are a number of third-party events, which are categorized into S, A, B, and C according to their size, influence, and the level of the teams participating (in descending order). Various event brands undertake independent event planning, and each tournament's results affect a team's ranking in terms of real-time credits with tournament weighting.

#### 2.2.3 League of legends (LOL)

LOL is a highly competitive game with divisions based on regions. The league system is carefully organized with spring and summer tournaments to keep players engaged and excited. The Mid-season Invitational (MSI) tournament is held in the middle of each season and draws in players and audiences worldwide. After the competition season has finished for a particular year, the global finals take place, with the top club in each division being selected. LOL has been selected for the Asian Games, where players from various countries form national teams to compete at the highest level.

#### 2.2.4 Honor of kings

*Honor of Kings* has emerged as a popular mobile esport, with the Honor of Kings Professional League (KPL) serving as the main league. The KGL (KPL G-League) acts as a feeder league for the KPL. Alongside this, the King of Glory World Champions Cup (KCC) is the highest-specification professional championship. This is held every summer and winter and attracts large numbers of players and spectators, especially from Asia (27).

Popular esports events within a mature tournament system are subject to a clearly defined event classification system. They are administered by either a tournament league or tournament committee and are held at various levels throughout the year or season, with specific fixtures and schedules. Given no unanticipated problems, the schedule of the highest official competition is always fixed. The top third-party leagues are often more stable during competition seasons and have taken on their own identity as brands. Smaller competitions are usually scheduled for when no top league events have been planned (28). Esports players have a demanding schedule that involves training, competitions, and commercial activities. Training is the most time-consuming activity (29), with a professional esports player spending an average of 10–12 h every day on improving their personal skills and team cooperation. Clubs arrange the players’ schedules for the competitions. During periods with more competitions and playoff matches, the pressure can be very challenging (30). Athletes and teams are subjected to high intensity repetitive training to meet the demands of top-level competitions. Players must be in excellent competitive condition for clubs to obtain high scores in events. As a result, tournament committees have taken to declaring an off-season and breaking up longer seasonal competitions into smaller ones to help the club players maintain excellent levels of competitive fitness. The off-season also gives clubs an opportunity to adjust their squads in preparation for subsequent matches. For example, CS:GO specifies one month each for its summer and winter off-seasons. The League of Legends has an off-season during the middle of the competition period and after the World Championship. No competitions are scheduled during an off-season and clubs provide active players with time to rest. However, it is during this period that the transfer market becomes most active as clubs seek to review their squads according to their previous performance.

Most of the participants in popular esports competitions are represented by clubs. These clubs consist of players, coaches, and other professionals associated with managing the club's participation in major events. The club's primary responsibilities include organizing competition participation, player selection, team training, personnel health management, and life service guarantees. Ensuring the smooth operation of the core business is essential for a club to participate in competitions and win prizes. A club's most valuable resources are its players and coaches. Selecting professional players, conducting transfer transactions, and initiating renewals constitutes a significant expense for any club. A well-qualified competition squad offers a range of possible rewards. At the same time, a failed squad can result in unpredictable losses. Forming a high-quality lineup is therefore closely bound up with the investment of club funds. However, there is no guaranteed positive correlation between investment and club performance. There have been numerous instances of high investment with low rewards and, contrariwise, low investment leading to high rewards in various esports projects (31).

### 2.3 The transfer market

Esports clubs aim to achieve excellence in competitions while generating income through strategic operations. The success of a club hinges on its ability to maintain a competitive edge, which is heavily influenced by the transfer of players. The player trading market is a dynamic ecosystem where clubs must balance cooperation and competition to thrive. Clubs usually change their squad according to past performance and budget for each new season. They can choose players to sell or buy within the market to try and meet target objectives, depending on quotations from other clubs. Junior players and players without contracts are often prioritized for clubs with tight budgets and changing needs. Some clubs with junior rosters will also consider promoting players from their youth squad to the main team. The more expensive players are on the market, the more likely they are to be active club players. In that case, clubs often have to pay high buy-out fees to make them switch clubs and very expensive players may not move quickly on the transfer market (32). Once players are identified for purchase, they will be tested in a trial session where they meet the new team. Players who pass this test will contract with the buyer's club. Therefore, the buying club, selling club, and player must abide by a confidentiality agreement that governs the transaction until the club or league announces the transfer results. This process protects the market and the players’ rights and interests. The tournament committee, typically led by a game manufacturer, plays a vital role in all of this. As a result, cooperation and competition have to coexist between clubs in the player trading market.

### 2.4 The role of information

Esports information plays an essential role within the industry and has to cater to a broad audience. Prior research suggests that esports information can be broadly categorized into three types: information regarding competition events; professional information; and transfer information. Information regarding competition events is the most important aspect of this and is released mainly by event organizers, who have a particular focus on events. Professional information in the field of esports is developed by related practitioners who specialize in dedicated interests such as game content creation, media interviews, data analysis, video creation, and esports activities. Transfer information, however, which relates to player transfers or club roster changes, has a variety of potential information sources, including the official competition league, professional media, and personal media. As player transfers are subject to confidentiality agreements and information about them typically needs to await announcement by official sources, verifying the authenticity of any other information about them in professional and private media is potentially difficult (33).

Traditionally, the transfer of esports players has been conducted according to the rules of the tournament federation or committee. The information provided in the official tournament or club announcements is the primary source of information for all parties regarding the transfer. The information provided in the announcement is an accurate statement of the outcome of the completed transaction (34). As transfers take place during the off-season and are subject to a confidentiality agreement, it is almost impossible for all parties involved in the transaction to announce transfer information to audiences before the contract is finalized. Additionally, there is a delay between negotiation of the transaction and signing of the contract. As a result, most transfer deals are not announced until the end of the off-season. These two factors have led to a lack of one of the key kinds of information of potential interest during the off-season. This can have an impact on the engagement of both esports players and spectators.

With the advent of social media and the advancement of the esports industry this situation has started to change and a growing number of esports informants have appeared who can service the thirst for information amongst the audience and esports professionals (35). These informants have relevant expertise in esports and are primarily focused on collecting and disseminating esports information. This is often released through personal social media or professional media channels (36). Analysis suggests that the career experience of these esports insiders is similar to that of traditional sports informants, namely, that they have worked previously, or are still working in the industry. They include previous professional competitors, club operating staff, and media esports staff. Importantly, esports informants are often closely related to the principal parties involved in transfers, i.e., competitors and club staff.

Esports is a rapidly growing industry and the nature of esports reporters as new practitioners and their impact on the esports information ecology is a relatively new research topic. Some existing work has already focused on how esports industry reporters might best cater to the audience (37). As a result, the demand for skilled and knowledgeable esports reporters has increased (38), making it a promising career path for aspiring professionals. However, despite the growing importance of this role, there has been little research on the impact of esports reporters on the industry. This study aims to fill this gap by examining esports reporters’ professional position in relation to the dissemination of information within the industry and the impact of their behavior on the broader media circle. While there may be limited research on the subject matter within esports itself, some related studies are still available for reference. For example, a 2021 Spanish study looked at the role of breaking news in relation to football and found that this kind of media content had a definite impact on the industry and its audience. This study is a prime example of scientific and systematic research on the relationship between sports and internally informed media. A number of studies of esports have turned in the first instance to traditional sports research findings (39–41). Indeed, the thriving sports media industry, particularly in football, is bound to offer certain lessons regarding the future development of esports media. The perceived value of this kind of research in other sports domains also serves to validate its potential value to the esports industry, underscoring the potential significance of the questions being explored within this paper (42).

## 3 Research objectives and questions

As noted above, over the course of its development, esports reporting has emerged as a specific profession. Informants working in this capacity play a particularly valuable role when it comes to the dissemination of transfer information during the off-season, filling what was previously an information gap in the industry. The study reported here therefore had three principal research objectives: to analyze the dynamic development of esports informants in the esports information industry; to analyze and compare audience engagement with information published by esports informants and its impact on the structure of off-season news; and to examine esports informant professional development and its impact on audiences and the esports industry.

To achieve the above objectives, we formulated four research questions:

**RQ1**: What was the quantity and proportion of information released by esports informants from 2013 to June 2023?
**RQ2**: How did the popularity of transfer information evolve from 2013 to June 2023?
**RQ3**: What was the average difference in the amount of information released between each off-season and competition season from 2013 to June 2023?
**RQ4**: Did esports transfer news attract greater levels of audience attention during the off-season than during the competition season?

In the next section, we describe how we set about addressing these questions by using one of the most popular esports as a case study.

## 4 Materials and methods

The general goal of the work reported here was to clarify the position of esports reporters in the industry and the impact their information has on the industry and its audience. To do this, CS:GO was taken as a specific case study and information data from its inception as an esport in 2013 to the first half of 2023 was assembled to underpin an approach founded upon content analysis.

While most of the popular esport team games have an active transfer market, there are a number of reasons why CS:GO was chosen for the case study. Esports such as Dota 2 and LOL have a large player base and complete competition systems, but they lack well-functioning cross-regional international teams because of some inherent limitations in how their regional competition systems operate. Basically, there are many differences in the rules according to the competition area. During the offseason, they therefore tend to focus on transfers within their regional division, and international transfers are relatively scarce (43). Meanwhile, over the past decade, CS:GO has become a dominant force in esports, with it steadily rising to prominence between 2013 and 2023. The game has its roots in the highly successful CS1.6 project and has inherited a well-established event system. HLTV.org serves as the game's official event information portal (44). Thanks to the rich data samples from the previous project CS1.6, CS:GO has established itself as a mature and reliable platform for competitive gaming. CS:GO's competition system is also relatively mature. The MAJOR is its most significant official event, but there are also third-party events. Its international club system allows teams to be selected globally. Its popularity as a competitive sport has also resulted in it already being the subject of academic attention (45). The internationalization of CS:GO's tournaments and clubs has allowed professional players to compete easily worldwide because the governing committee has eliminated regional restrictions on transfer, resulting in international teams and players transferring between regions (46). The team composition guidelines require a minimum of five competitors and a coach. This has led to a sizeable and well-developed transfer market that offers a source of abundant data for this specific project. Clearly, a study of the information ecosystem in other esports would provide valuable additional depth to the work presented here. However, in view of the characteristics of CS:GO, especially its well-developed international transfers market, this case study is already able to provide a number of useful insights into the importance of esports reporting and the value it brings to the industry. In view of the extent and clearly scoped nature of the data available and its capacity to address our research questions, we would also argue that using content analysis to investigate the information ecosystem in CS:GO more than adequately meets the definitional and boundary requirements proposed by Yin (47) with regard to the conduct of effective case studies.

**Figure 1:**
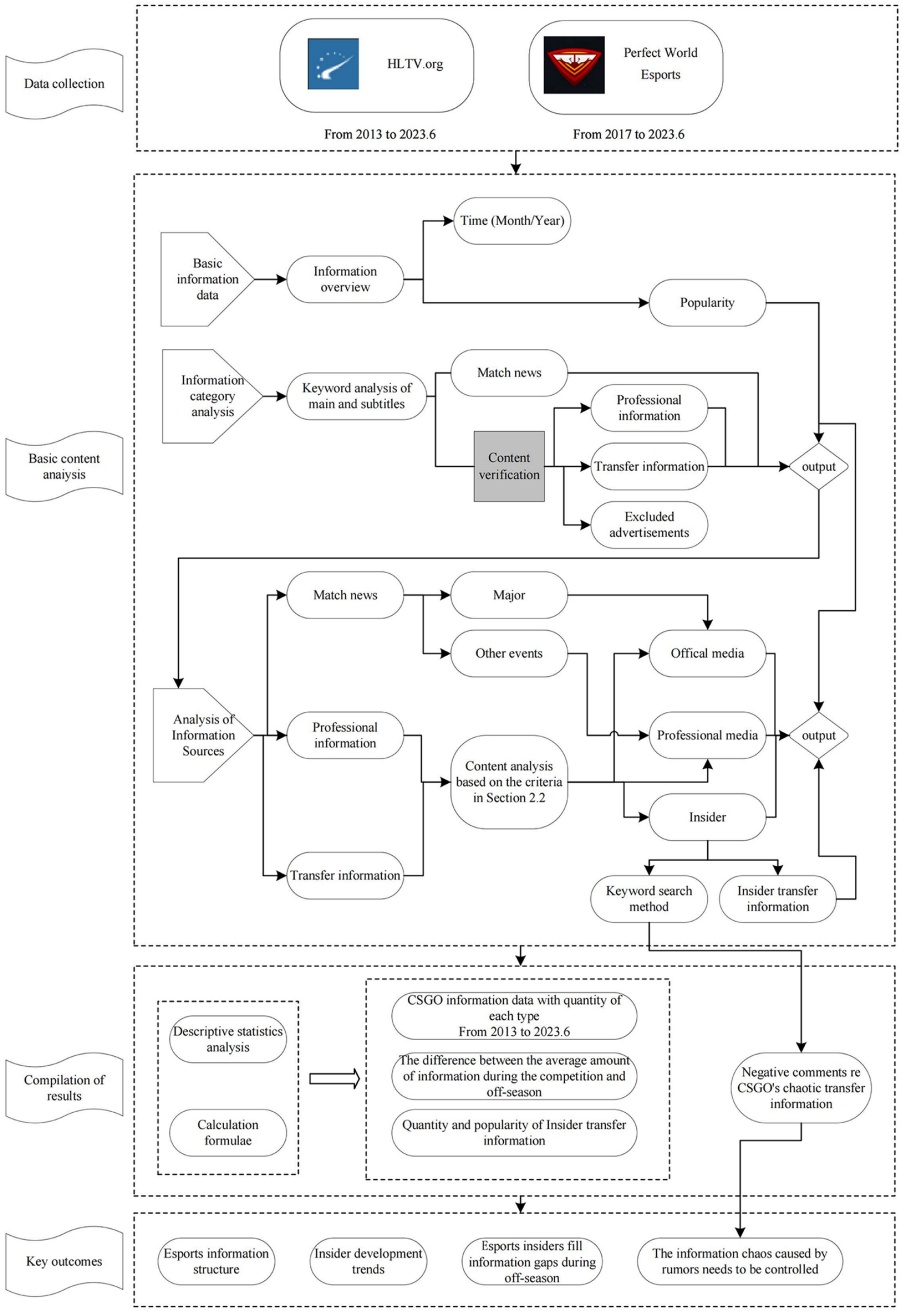
Research process.

An overall depiction of the research process is shown in Figure 1. We describe the process in greater detail below.

### 4.1 Data collection

The study data was taken from Counter Strike news, the esports coverage website HLTV.org, and the Perfect World Esports APP. These platforms deliver information to game officials and game practitioners and provide professional news updates. Player-viewers also have the opportunity to leave comments on the platform, which serves as a further potential source of data. Generally, these official platforms are dedicated to ensuring professionalism and the traceability of information.

HLTV.org is the leading Counter-Strike coverage site in the world. With on-site coverage of all major tournaments, it features HLTV, GOTV, stats, demos, news, results, rankings, videos, photos, and more (48). Esports information was retrieved from *the News* site of HLTV.org, where the sidebar makes it possible to find Match news for a specific year/month or according to a designated tournament. Through the news search for a selected month, one can browse by title, source location (country/region), post date, and the number of comments. The detailed page for each news item contains the title and subtitle, the name of the editor, release time, information content, relevant links, links to external sources, and comments.

In the Counter Strike section in Perfect World Esports APP, the platform allows one to find news by using keywords. The overview page for the news items contains the title, overview pictures, release time, category, the number of visitors, and the number of comments. The detailed page for each news item contains the title and subtitle, the name of the editor, release time, information content, source statement, keywords, and comments.

As can be seen in Figure 1, data was collected from HLTV for the period from 2013, when CS:GO started to operate, to the first half of 2023. The data collected from the Perfect World Esports APP was for the period from 2017, when the platform was established, to the first half of 2023. After comparison and verification, some news items were found to have been posted on both platforms. In these cases, we used the information from HLTV as the statistical source.

### 4.2 Content analysis

Content analysis covers a wide range of different techniques and approaches. Originally it had a tight linguistic focus on the use of certain words in texts, but these days virtually any form of textual analysis that concentrates on how texts are constructed and/or used may be considered content analysis (49). Obviously, as the number of different types of texts available via the internet has proliferated, content analysis has come into its own as a way of understanding the nature of those texts and how they operate (50). Broadly speaking, there are three kinds of content analysis: conceptual analysis; procedural analysis; and relational analysis (49). Conceptual analysis focuses on the concepts used in a text; procedural analysis focuses on how the content of a text may be said to perform some activity; and relational analysis is more interested in the underlying mental models that inform the construction of the text (49). There is also a distinction between quantitative and qualitative content analysis. Quantitative content analysis focuses on counting things like words and categories within a collection of texts, while qualitative content analysis is interested in how different texts might be interpreted (51). In this study, the content analysis is of a quantitative character and may be said to be primarily procedural in its form.

A content analysis (52) of news relating to CS:GO released on the Perfect World esports APP and the HLTV website between 2013 and June 2023 was undertaken to form the basis of the case study. The data from the official esports news for the period between 2013 and the first half of 2023 was organized in chronological order. First of all, we focused on data regarding the release date and popularity of each news item. The overall time period covered by the research was divided into a year/month time series. The content of each news item was analyzed month by month from 2013 onwards and the popularity of each news item was recorded afterwards. A worksheet was created to guide the content analysis of each item of news in the sample. This was organized in terms of: Time (Year; Month; Major; Off-season); Information Type (Match News; Professional Information; Transfer Information); Information Source (Official Media; Professional Media; Insiders); and Other (e.g., Insider Transfer Information). In relation to this, the published units of content were selected and categorized according to a number of criteria. For selection, the content had to be related to the game, esports, and virtual game accessories. We excluded advertising delivered by gambling websites and peripheral merchants. After that, the information was first arranged under three headings: publisher; title; and textual content. Annotated reprints were then classified according to the source of the informational content. Authors were identified as official if they released major tournament event information. Except for the major tournaments, information released by event-organizing media was classified as content released by professional media. Information about player transfers or franchises coming from individual players, insiders, personal media in the industry, and internal whistleblowers the club did not license was all treated as information from personal whistleblowers. Where the same content was published on different media platforms, it was treated as just one piece of information and the corresponding popularity value of the information was taken from the platform with the highest popularity value. The transfer results for a specific player, issued by a club or official media, were subject to an announcement of completed lineup changes. The popularity value of the information was calculated differently for different network platforms. Overall, the value of the information was selected according to its authenticity and comparability. Beyond this, months containing Majors were marked as Major months. If the Major spanned two months, the month covering the larger number of tournament days was selected as the Major month.

Once the individual online news items had been analyzed, Microsoft Excel was used to organize and process them so that the overall data could be analyzed in greater depth. After that, the time ordered data was further divided up using the keywords from the title and subtitle to achieve pre-classification. Inspection of the information content then helped us to estimate the accuracy of the pre-classification. At this point, posts focused on betting advertising and computer peripheral advertising were excluded, while Esports club advertising, sponsor contract signing, and event organizer advertising were classified as Professional Information.

Finally, a comprehensive analysis was conducted that covered keywords in the title, the editor, the content, and the source links. Three categories were defined that divided the content up into official media, professional media, and insider information, according to the information sources. Because of its centrality to the study, transfer news from insiders was independently listed.

It should be noted that, before 2015, the off-season lacked precise definition; it occurred after the Major at the end of one year and the beginning of the next year. After 2015, the off-season was jointly determined by all major competition parties. Additionally, it will be seen that the data for 2020 was affected by COVID19 and was significantly different from other years [see (53) in this regard].

On the basis of the classification of the different news items, Supplementary Table S1 was created. In Supplementary Table S1, the Total is the sum of the match news, professional information, and transfer information. The Popularity figure is the sum of the number of comments relating to this type of information received during the month. The Proportion is the proportion of this type of information in relation to all of the information published during the month. The Insiders Transfer Information is the quantity of transfer news items produced by insiders. From the data in Supplementary Table S1 it is possible to deduce things like: the quantity of the three different types of information per month and per year; the number of items coming from the three different information sources per month and per year; the popularity of the three different types of information per month and per year; the popularity of the three different information sources per month and per year; the quantity and popularity of the transfer information produced by insiders; and the timing of the off-season and the Major for each season.

In the results presented below, the paper focuses on the data relating to the information sources and types of information. Alongside this, time was set as a variable and the browsing popularity was used as a criterion for comparison. By using this approach to analyze the content, we were able to address each of our research questions.

## 5 Results

Supplementary Table S1 (see the Appendix) comprises the complete dataset, with a total of 26,278 pieces of information from HLTV.org and Perfect World esports. The data collected relates to the period from 2013 to mid-2023. As noted above, the types of data cover whether the information was match news, professional information, or transfer information.

**Figure 2:**
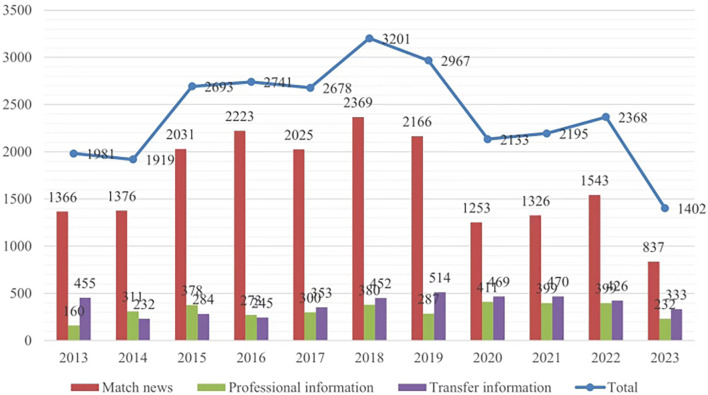
Counter-Strike: global offensive (CSGO) information data.

Figure 2 displays the quantity for each classification annually and the overall total. The bar chart indicates the amount of Match news, Professional information, and Transfer information for the 12 months of every year. The total information, which is displayed as a flow chart, shows the total number of all three information types for the corresponding year.

**Figure 3:**
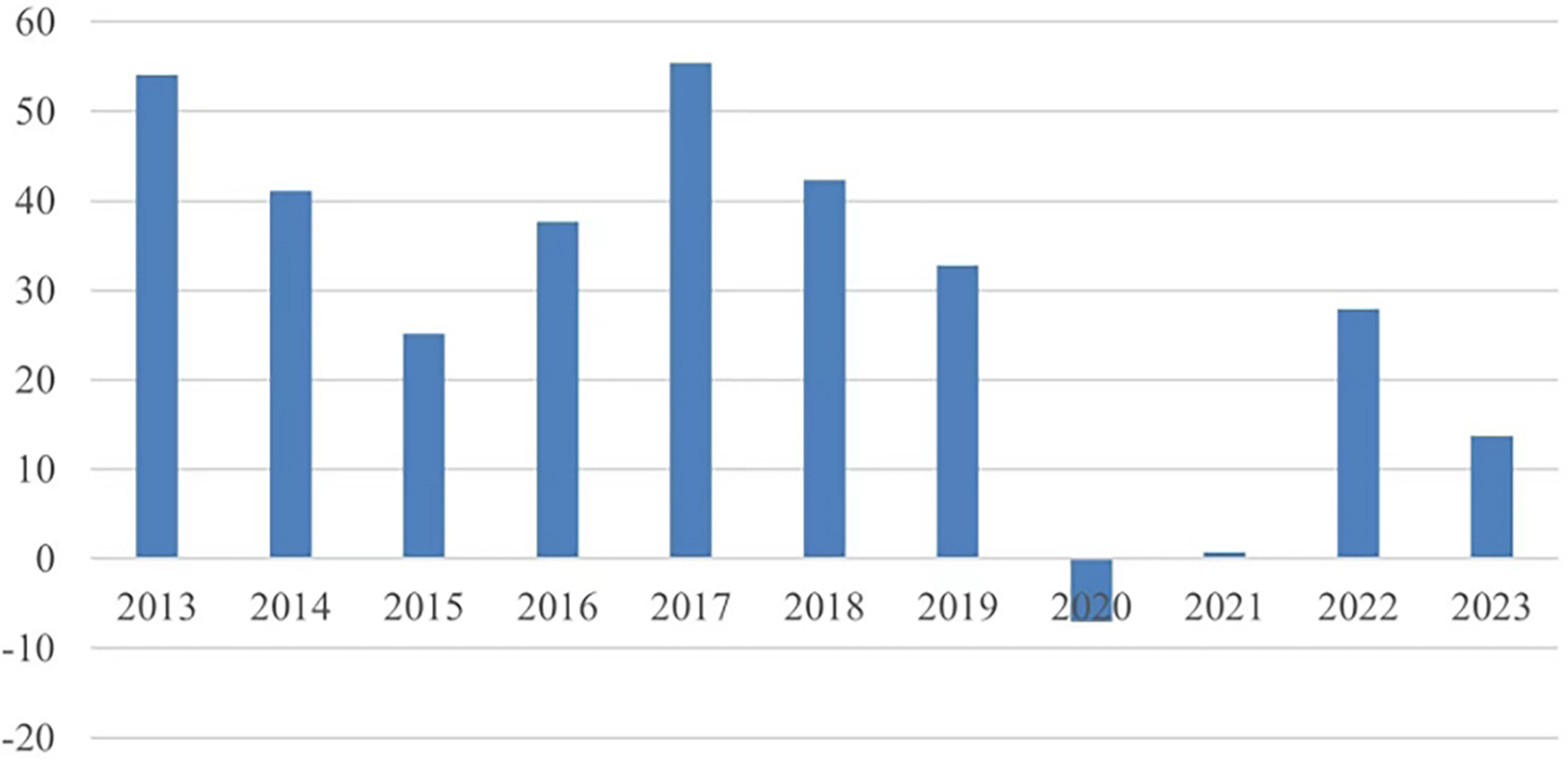
The difference between the average number of news items during competition and off-season periods.

Figure 3 shows the difference between the average amount of information during the competition and off-season periods. The numbers in the bar chart were calculated from the total amount of competition news every year divided by the sum of the competition news months of that year minus the total amount of off-season news every year divided by the sum of the off-season news months for that year.

**Figure 4:**
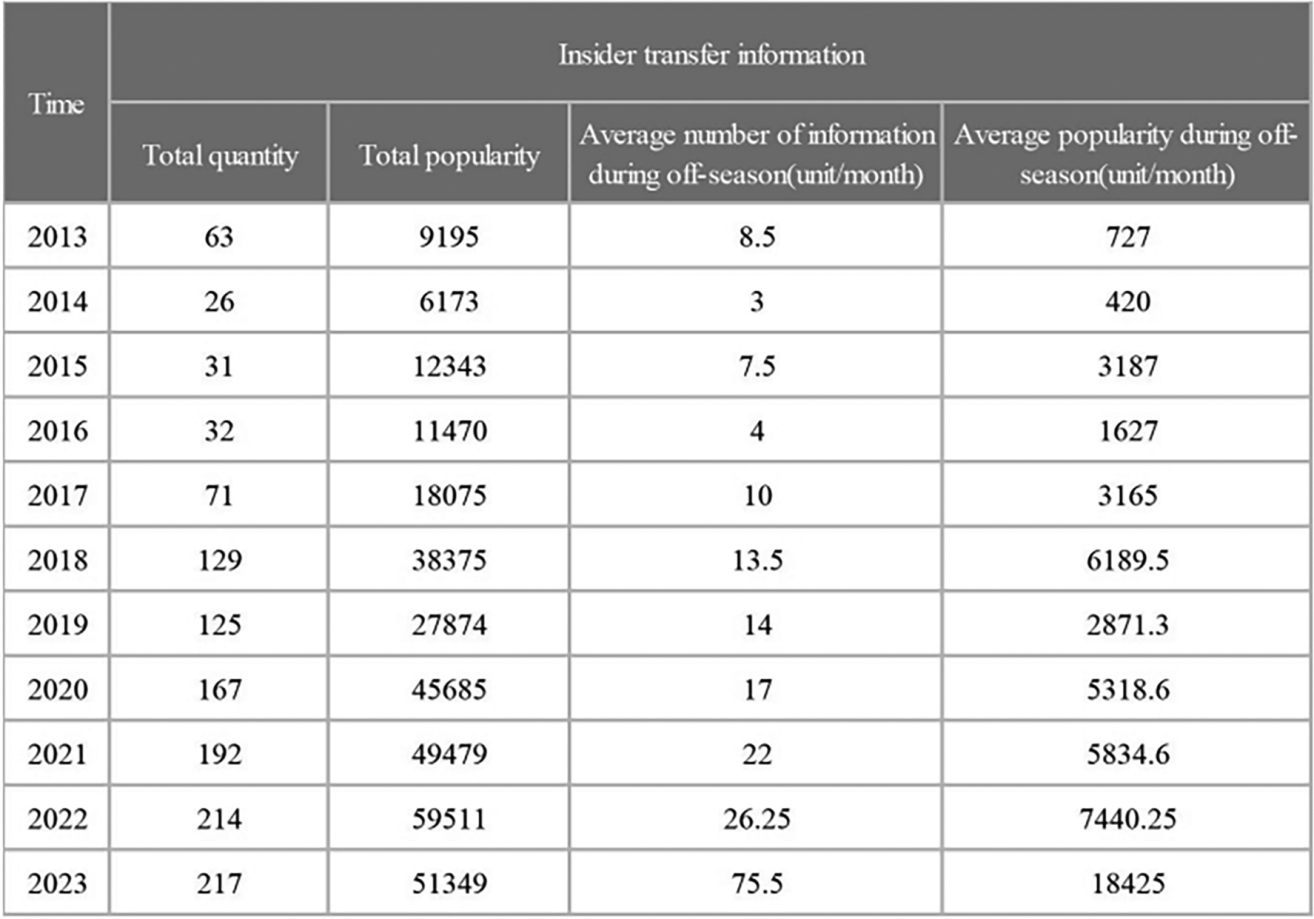
The impact of insiders on the dissemination of esports information.

Figure 4 provides a more intuitive illustration of the impact of insiders on the dissemination of esports information. The figure shows the total amount and popularity of transfer information reported by insiders each year. Statistical analysis was conducted on the monthly average amount of information and the monthly average popularity of insider transfer information for each off-season.

### 5.1 Volume and proportion of information coming from esports informants (RQ1)

As shown in Figure 2, out of the total amount of information (*n* = 1981), the highest proportion of transfer information (23%, *n* = 455) was reported in 2013. This higher starting point was because the professional players had to adapt to the new version of the game and competition as it changed from Counter-Strike:1.6 or Counter-Strike: Source to CS:GO. This process led to a large number of player transfers during that period. From 2014 to 2019, the proportion gradually increased from 232 to 514 because of the increased frequency of events, which drove an overall rise in the amount of event information year by year. The proportion of professional information remained stable from 2014 to 2018. In 2019, there was an increase to 17.3%, then to 21.3% in 2020, and it remained above 20% from 2021 to the first half of 2023.

The volume of information in the esports industry has generally increased since 2019. In 2020, COVID-19 led to the cancellation of many esports events, resulting in a decline in competition events to 58% of the figure for the previous year. The offline games resumed toward the end of 2021 and the volume of information since then has been growing yearly. By the first half of 2023, the volume of information had returned to pre-pandemic levels. Setting aside the club reorganizations that occurred as a result of the change of game versions in 2013, the volume of information has followed a consistent growth trend. This growth in the volume of information reflects the overall development of the esports industry. As a result, while the information structure has continued to be based on match information, it has gradually become more diverse as interest has grown in a wider range of associated topics.

### 5.2 Difference in the average amount of information between the off-season and competition season (RQ3)

As can be seen in Figure 3, the difference in the overall amount of information released between the off-season and the competition period gradually narrowed from 2013 to 2015. However, the difference increased again in 2016 and 2017 due to an increase in the frequency of competition activities before falling off again because of an increase in the amount of information released by informants during the off-season. Generally, referring to Supplementary Table S1, the proportion of information released during the off-season when measured against the whole year is lower than that of the competition period because of the lack of competition activities during the off-season. The volume of information during the competition period is largely stable, but the number of competition activities significantly reduced in 2020–2021 as a result of COVID-19. Thus, the amount of information released in the 2020 off-season was actually higher than it was in the competition period, and the difference remained close to 0 in 2021. It is evident that the frequency of competition activities dramatically affects the volume of information during the competition period. However, the COVID-19 pandemic had less impact on the volume of off-season information, when there was a higher proportion of transfer information. Although there was a slight increase in the difference between regular and off-season transfer information after the gradual resumption of sporting activities, it was still lower than the levels observed in 2019 and earlier. However, from 2022 onward, the amount of transfer information during the off-season shot up again as the audience sought to redress the information gap. Since 2017, the regulations set by the CSPPA have ensured that the CS:GO tournament is well-organized and competitive. From the middle and end of 2022 to the beginning of 2023, CS:GO had a uniform off-season that lasted for one to two months after the end of the official World Class event, making it the perfect time to take a break and prepare for the next season.

### 5.3 Popularity of transfer information (RQ2, RQ4)

The popularity of transfer-related information has increased since 2019, with an explosive growth in the number of comments. Our results relating to this are shown in Figure 4. Since 2020, the number of comments per year has never been less than 45,000, reaching 51,349 in the first half of 2023 alone, which almost matches the total number for 2022. Prior to the epidemic, the number of comments about transfers had increased to 12,974 in 2018, the most populous year before the event. This surge in popularity can be attributed to an increase in transfer market activity and a corresponding increase in information released by reporters. With the lack of games during the off-season, the audiences have shifted their attention to this kind of content and the number of informants has increased to meet the demand.

The specific timing of transfers is adjusted according to the scheduled events. Nonetheless, the off-season is a significant period for clubs to switch players, resulting in an increase in transfer-related information when compared to other months. A credit ranking is used to qualify for entry into the popular esports events in the official tournament. As a result of this system, the clubs have to earn enough credits in the preselection events with a specific team lineup. Changes to the lineup can lead to them losing some of their credits.

According to the data we collected, the proportion of transfer information in the competition months decreases once the competition fixtures have been established. After the official tournaments, there is typically an off-season where the esport’s popularity declines and the circulation of professional data reduces. During this time, the amount of data generated by the events also reduces as no new games are being played. As the off-season coincides with the transfer period it is not surprising that the proportion of off-season transfer information increases in comparison to other months in the same year. The difference between the average amount of information during the off-season and during the tournament period was larger before 2018, after which it decreased. Thus, it would appear that the off-season has gradually become a time where the information is primarily focused on transfers.

As a final point, within the collected data, the number of insider informants increased yearly, with the growth rate accelerating after 2020. The information popularity at the end of the study in the first quarter of 2023 was already close to the figure for 2022. Transfer information from insiders is, of course, less accurate than that of official and professional media. However, uncertainty surrounding the accuracy of the transfer information can itself generate discussion and audience interest, making the information more popular and generating yet more buzz.

## 6 Discussion

### 6.1 Impact of insider information on the esports industry

Our results have shown that, with the growth of the esports industry, insider informants have emerged to cater to the audiences’ desire for news when other information is hard to come by. This forms part of a general move towards independent reporting in esports news (54). These insiders provide transfer information that can itself have an effect on parties to the transfer, such as professional players and clubs. In the materials we gathered, we found that, since 2020, discussion about these informants has begun to appear on official and professional media platforms. We also saw professional players, club administrators, and esports practitioners expressing their own opinions regarding the informants and their releases.

Transfer transactions revolve around the players and have a significant impact on the earnings and future development of professional esports players (55). Before the official announcement of a transfer results, informants often warn the audience that what they know about a transfer's content, progress, and details cannot guarantee the information's authenticity [see, for instance, the disclaimers on Liquipedia's transfer news portal (56)]. Judging by the proliferation of responses to certain transfer stories in our data, the most common outcome of such speculations is to arouse heated discussion within the audience. We also saw a further proliferation of responses after the official announcement of transfers.

Professional players may respond to the released information if it is inconsistent with what they already know or may have a negative impact on the transfer [see, for instance (57),]. As competitors are the main source from which insider informants acquire their information, it seems likely that their behavior will have a major influence on their future relationship with competitors. In other words, inaccurate and harmful reporting by informants may jeopardize their capacity to acquire further information from the same sources. Thus, there are some checks present on how many liberties informants may take with the facts. Nonetheless, many well-known professional players (e.g., Russel “Twistzz” Van Dulken (58); Aleksandr “KaiR0N-” Anashkin (59); Nikola “Niko” Kovač (60); Jesper “JW” Wecksell (61); and Peter “dupreeh” Rasmussen (62)) have made negative comments about the CS:GO media's chaotic handling of transfer information via their personal social media and in HLTV interviews. Clearly, the risk of false rumors about high-impact topics such as transfers stands as a potential source of concern for the esports industry. Nonetheless, few clubs seem to actively seek to quash transfer leaks, so it would seem that they accept it as an inevitable part of the enterprise. Indeed, in view of the attention that can be garnered amongst audiences through speculation about transfers, one could argue that such disclosures have a commercial value.

### 6.2 Insider information trends in the esports industry

The esports industry is large. Like traditional professional sports, rumors and transfer leaks are part of its information ecology and business. Speculative transfer information is bound to appear in the absence of any other information and its disclosure plays a stimulating role in esports circles. However, differences in the degree to which it is professionally handled can affect the esports industry positively or negatively. This has to be offset against the thirst of audiences for currently undisclosed information. It is important to note that social media and esports have grown alongside one another in recent years, so not all insiders providing information are necessarily credible practitioners, though audiences will sometimes take such journalists to task [see (63)]. Even just a cursory search on “twitter esports journalists” reveals that there has been a blossoming of X (previously Twitter) journalists in the esports information industry in recent years. This makes it imperative that the insiders providing information should adhere to high professional standards to protect the future development of the esports industry (64). As a part of this, preliminary assessment of the information collected should be done before any disclosure. As the content is intended to meet the needs of both the audiences and the promotion of transfers, regulating its release can reduce any adverse impact that leaks may have on the actual transaction and involved parties. In view of the media channels it is typically exploiting, insider information can potentially have extensive reach, making it of unique commercial value. Our findings show that, as esports have become more specialized, information disclosure around topics such as transfers has become the main content “filler” in off-season periods when no other information is available. This is bound to attract the attention of audiences. In the face of this, the role of “insider informant” would appear to be gradually evolving into a recognizable profession, providing esports practitioners with a potential new career path. This merits careful monitoring by both the industry and research as it continues to develop.

## 7 Conclusion and limitations

To sum up, insiders have been instrumental in filling in gaps in the available information during off-season periods in the esports industry. Breaking information from such sources has provided audiences with valuable insights into the esports transfer market. The study reported in this paper set out to explore how the role of esports insider informants was developing, how audiences were engaging with their output, and how their role was evolving into a recognizable profession. The results have shown that the popularity of transfer information has increased since 2019. Given this increase in attention, the breaking news during the esports off-season has gradually matured, enriching the esports information ecology. We have seen that insider informants have come to play an increasingly important role in this ecology, and this has implications for their position and status within the esports industry. As an emerging profession, esports reporters offer significant added value by filling what would otherwise be a problematic gap in the information cycle. On top of this, it is important to note that this new profession can also provide career transition options for professional esports players and personal media practitioners. In the future, it may even make sense to introduce media learning materials into esports education.

Nonetheless, while the materials we have presented here provide valuable insights, the study also has several notable limitations. In particular, despite its mainstream status, CS:GO has a competition system and information structure with certain particularities. Therefore, some of the phenomena discussed in this article may not apply to other esports projects. We would therefore acknowledge the importance of similar studies being undertaken for other esports, so as to build up a more comprehensive corpus of data. We also acknowledge that the quantitative focus of the research reported here is restricted in the amount of insight it can offer. It is therefore important that further qualitative research be conducted into how insider reporting is conducted and its impact. In-depth interviews with esports practitioners, such as professional players, professional commentators, analysts, club managers, and tournament committee members would help to generate more conclusive and actionable results. By tapping into the views and experiences of industry insiders, it would become possible to draw more specific conclusions regarding this aspect of the esports industry, particularly regarding the prospects for the professionalization of independent and insider esports reporting. We therefore strongly advocate further research and interviews with relevant parties to gain a more comprehensive understanding of the topic. Such interviews may also generate valuable insights into where industry insiders themselves feel research might best be directed. More broadly, we believe it is crucial to conduct case studies across a range of topics to gain a better understanding of the esports industry. These studies should cover the industry's development, the esports information infrastructure, and the role being played by consulting practitioners. Only by undertaking these kinds of studies will it be possible to acquire the kind of in-depth understanding required for research to provide useful and actionable insights that can support and assist the future development of the industry.

Having recognized that the study reported here only represents a first step in the development of a rich body of insight, it is important to remember that, even if it is partial, there are a number of ways in which distinct esports have developed in line with the overall development of the esports industry, including a burgeoning transfer market. Therefore, the results of this particular study are likely, at least in certain respects, to reflect developments in the dissemination of information across the entire esports industry. They may also serve as a source of guidance and inspiration for research dedicated to other esports-related projects.

## Data Availability

The original contributions presented in the study are included in the article/**Supplementary Material**, further inquiries can be directed to the corresponding author.

## Supplementary material

Supplementary Table S1 Counter-Strike: Global Offensive (CSGO) information data from 2013 to the first half of 2023.
